# Sleep-Wake Behavior in Elite Athletes: A Mixed-Method Approach

**DOI:** 10.3389/fpsyg.2021.658427

**Published:** 2021-08-03

**Authors:** Kévin de Blasiis, Hélène Joncheray, Julia Elefteriou, Chloé Lesenne, Mathieu Nedelec

**Affiliations:** Laboratory Sport, Expertise and Performance (EA 7370), French National Institute of Sport (INSEP), Paris, France

**Keywords:** actigraphy, training, sleep environment, interview, qualitative data

## Abstract

**Purpose:**

Using a mixed-method approach, this investigation aimed to examine athletes’ sleeping patterns along with the socio-physiological acute and chronic stressors affecting their sleep.

**Methods:**

Fifteen elite athletes (*M*_*age*_ = 21.7 years; *SD* = 2.2) were monitored daily during a typical in-season training period (20 ± 1 days) and completed individual semi-structured interviews. Their sleep was analyzed using actigraphy and sleep diaries. A content analysis method was used to assess qualitative interviews.

**Results:**

Three factors influencing sleep emerged from the qualitative interview analyses, i.e., sleep environment, training and study requirements. Half the athletes (8/15) complained about their sleeping environment with noise and/or bedroom temperature and/or mattress quality and/or light exposure presented as an issue. “Complainers” notably exhibited impaired (*p* < 0.05) sleep efficiency and wake after sleep onset compared with “non-complainers.” Daily training load showed correlations (*p* < 0.05) with wake after sleep onset and sleep onset latency. “Student athletes” exhibited later bedtime and earlier wake-up time compared with “non-student athletes,” leading to a reduced total sleep time (6:50 ± 1:13 vs. 7:06 ± 0:53; *p* < 0.05; *d* = 0.20).

**Conclusion:**

An individualized assessment of sleep using actigraphy and interviews, with consideration to various socio-physiological factors, is recommended. Individualized sleep interventions with consideration to physiological (e.g., training load), behavioral (e.g., screen use) and environmental factors (e.g., room temperature, noise, mattress), can then be provided to each athlete.

## Introduction

Elite athletes are particularly prone to sleeping disorders. Due to many social and sport-specific stressors, recommending a generic sleep duration for athletes is difficult (Fullagar et al., 2015). Some authors recommend a sleep duration of 9–10 h per 24-h cycle for athletes (Bird, 2013) vs. 7–9 h for the general adult population (Hirshkowotiz et al., 2015). According to several studies, such recommendations are not achieved by athletes, with a frequently reported sleep duration of 5–8 h (Leeder et al., 2012). More than half of elite athletes suffer or have suffered from sleeping disorders without any compensatory sleep hygiene strategy for the majority of them (Juliff et al., 2015).

The importance of sleep on athletic performance, recovery and well-being has been consistently reported (Fullagar et al., 2015). Sleep is an essential part of the recovery process as it provides a number of important psychological and physiological functions, e.g., muscle glycogen repletion, muscle damage repair, cognitive function and mental fatigue alleviation (Nédélec et al., 2015). Although sleep is recognized by the majority of athletes and coaches as fundamental, there is currently a deficit in sleep monitoring among athletes which may be explained by a lack of resources and/or knowledge (Miles et al., 2018).

Studies which were conducted to monitor athletes’ sleep predominantly used actigraphy and, to a lesser extent, sleep diaries and questionnaires (Gupta et al., 2016). These tools are easy to use, descriptive in nature and answer the question “what” are we observing. However, athletes’ sleep is highly variable (Nédélec et al., 2018) with several sleep parameters more variable than age- and sex-matched controls (Leeder et al., 2012). Several candidate factors have been explored in the literature to explain sleep variability among athletes, e.g., sleep environment (Suetsugi et al., 2017), training time (Swinbourne et al., 2016), training load (Dumortier et al., 2018) and/or chronotype (Vitale et al., 2017). It has also been suggested that family structure, cultural environment, social pressure and/or use of media could have an important impact on sleep among the general population (Shochat, 2012). However, the latter factors are currently underreported among athletes. A better understanding of the socio-physiological stressors affecting the athletes’ sleeping patterns (e.g., early morning training session, use of media) could be of direct benefit to enhance recovery, and preparations for subsequent training and competition.

In order to encompass a broader range of stressors, a mixed-method approach was retained in the present study. A mixed-method approach is an interdisciplinary one combining different methodologies, e.g., objective assessment with qualitative interviews. It may be useful to investigate reasons put forward by athletes for not achieving sleep recommendations (Burlot et al., 2018; Joncheray et al., 2019). Using this approach, Nédélec et al. (2019) examined the link between sleep and injury occurrence in an elite male soccer player during 4 months. Only qualitative interviews allowed the player to explain the objective assessment of sleep. According to him, “match-induced nervousness and/or thoughts about the match” explained altered sleep after evening competitions; and regular afternoon medical care reduced the opportunity for napping. The level of evidence assigned to case reports is low and these preliminary results should be confirmed with a larger sample.

Using a mixed-method approach, the present investigation aimed to examine sleep-wake behavior in elite athletes in order to investigate athletes’ sleeping patterns along with the socio-physiological factors influencing their sleep. We hypothesized an important variability of sleep among elite athletes with both sociological and physiological factors responsible for this phenomenon.

## Materials and Methods

### Subjects

Fifteen international-caliber elite athletes (7 males, 8 females; *M*_*age*_ = 21.7 years; *SD* = 2.2) with a mean playing experience of 12.3 years (*SD* = 4.3) took part in the study. Exclusion criteria included athletes under the age of 18, unaffiliated to the French health care system and presenting health problems or sleeping disorders. All participants were involved in individual sports (modern pentathlon, *n* = 1; archery, *n* = 5; fencing, *n* = 2; athletics, *n* = 2; judo, *n* = 2; rhythmic gymnastics, *n* = 1; weightlifting, *n* = 1, figure skating, *n* = 1). Athletes were recruited via coach contact within the French Institute of Sport (INSEP), which is home to the top national athletes. Ten of them were students (13.2 ± 6.6 classroom hours per week) while the others did not have any work or school requirements. Data was collected during a typical, in-season training period (20 ± 1 days) free from competition. Mean ± SD weekly training volume was 23.5 ± 6.2 h. The study was carried out during the winter season (January to February 2019) at the French Institute of Sport (Paris, France), to minimize the effects of outdoor ambient light levels. All participants slept in the same dormitory. Prior to participation, they all signed informed consent forms and the protocol was approved by the local ethics committee (East III, France. Ref. 170605).

### Methodology

#### Daily Monitored Measures

Each participant received an activity monitor to record sleep data and a sleep diary in which to report daily subjective data. Actigraphy data was collected using the activity monitor worn on the non-dominant wrist (Camntech, Motion Ware 8, Cambridge, United Kingdom). The recorded data was scored automatically using Sleep Analysis software (Version 7.43, Camntech, Cambridge, United Kingdom), which indicated whether the participant was awake or asleep for each 60-s epoch. The actigraphy method has been shown to be reliable and valid for the objective measurement of sleep (Fuller et al., 2017). The medium sleep-wake threshold (≥40 counts of activity are scored as wake) generates the smallest mean biases compared with polysomnography for total sleep time, sleep efficiency and wake after sleep onset (Fuller et al., 2017). Given that a variety of sports was presently considered, a medium sleep-wake threshold was applied to the data set. Sleep variables were measured as follows: Time in bed (TIB) was defined as the time from lights off (bedtime) to the end of sleep; total sleep time (TST) was defined as the time spent asleep, as determined from the beginning of sleep to the end of sleep, minus wake time; wake after sleep onset (WASO) was defined as the total wake time, according to the epoch-by-epoch wake/sleep categorization; fragmentation index (FI) was defined as the sum of the mobile time (%) and the immobile bouts ≤ 1 min (%); sleep efficiency (SE) was defined as TST divided by TIB (expressed as a percentage); sleep onset latency (SOL) was defined as the time from lights off to sleep onset.

The rating of perceived exertion of the session was reported within 30 min of the end of each training session. Participants answered the question “How was your workout?” on a scale from 0 (rest) to 10 (maximal) (Foster et al., 2001). The answer was multiplied by the session duration (in minutes) to obtain the training load. All sessions on the same day were added up to obtain the daily training load.

#### Sleep Questionnaires

The Spiegel Sleep Inventory (SSI) was administered upon waking to assess the daily perceived sleep quality. The SSI is a self-administered questionnaire composed of 6 questions (score: 1–5) regarding sleep onset latency, sleep quality, sleep duration, nocturnal awakenings, dreams, and feeling refreshed in the morning. The global score is the sum of the 6 items. There is no available data on the psychometric validity of the SSI; however, it is a very simple and easy- to-use scale that is often used to assess the presence of insomnia (Léger et al., 2016). Additionally, participants indicated their level of sleepiness upon awakening on a visual analog scale. The participants made a mark with a pen to indicate their level of sleepiness, which ranged from 1 (very alert) to 9 (very sleepy with an important effort to stay awake) (Akerstedt and Gillberg, 1990). Participants also indicated if they used a screen (Yes/No) within the hour preceding bedtime.

The Pittsburgh Sleep Quality Index (PSQI) is a 19-item self-report questionnaire assessing general sleep quality and duration for the last month (Buysse et al., 1989). Seven component scores were calculated: (1) sleep quality, (2) sleep latency, (3) sleep duration, (4) habitual sleep efficiency, (5) sleep disturbance, (6) use of sleeping medication, and (7) daytime dysfunction. The global PSQI score range from 0 to 21. A score ≥ 5 was considered as a poor sleep quality.

The Morningness-Eveningness Questionnaire (MEQ) consists of 19 self-assessed questions, which determines an individual’s chronotype (Horne and Ostberg, 1976). The chronotype classification was performed as follows: 16–41 = evening type; 42–58 = intermediate type; and 59–96 = morning type.

#### Qualitative Interviews

**FIGURE 1:**
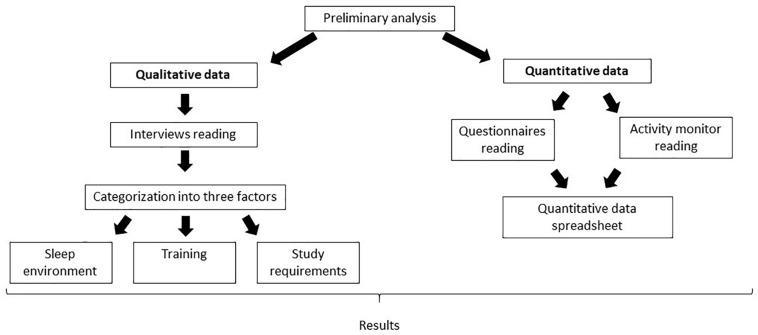
Methodological approach used to combine qualitative and quantitative data.

A qualitative interview can be defined as an interactional exchange of dialogue between two or more participants in a face-to-face context (Stroh, 2000). Providing a close and research-focused contact with participants, it allows for the collecting of a great deal of information through direct access to experiences, opinions and behaviors (Stroh, 2000; Rubin and Rubin, 2005). Semi-structured interviews, which are a more flexible version of the structured interview, were individually conducted and audio-recorded. Athletes were encouraged to talk about their experience with sleep, both as an athlete and a man/woman, from childhood to the present time. The sessions took place over 2 weeks in January 2019 in a relaxed and comfortable atmosphere in the presence of one or more researchers (KdB, JE, MN, and HJ). The issue of confidentiality was of critical concern; each athlete was given assurances that no negative impact from the research would affect their career progression. Each interview started with a summary of the athlete’s sports career, then the conversation addressed staff environment, training/competitions schedule, socio-professional issues before progressively focusing on sleep-wake behavior. Questions such as the definition of an “ideal” night’s sleep, sleeping patterns during childhood, the perceived impact of training/competition on sleep, the sleeping environment enabled the collection of specific individual information. Interview duration was 53 ± 14 min. The content analysis method defined by Krippendorff (2003) as the most widespread method used to assess qualitative observations, was used. The procedure consists of three steps: Transformation of an oral speech into a transcript (verbatim), information coding and data processing. Each interview was manually transcribed into a computer file. Then, an open coding system was used to divide the verbatim into themes. Finally, an empirical analysis was used to process data consisting in analyzing themes (synthetic stage), searching for explanatory factors (research stage) and evaluating these factors and key ideas (evaluation stage; Figure 1). After the analysis, the population was split into “complainers” and “non-complainers” of their sleeping environment if at least one of the following items was presented as an issue by the interviewee: Noise and/or bedroom temperature and/or mattress quality and/or light exposure.

### Statistical Analysis

A statistical analysis was performed using JASP 0.14 (University of Amsterdam). The normality of the data distribution was assessed using the Shapiro-Wilk test. Differences between groups were tested for significance using Student’s *t*-test or Wilcoxon unpaired test, depending on the normality of data distribution. The coefficient of variation [CV; CV = (standard deviation/mean) × 100] was calculated for the whole group and individually for sleep and training load over the study period. Within-subject correlations between sleep metrics and training load were analyzed using the Pearson product-moment correlation coefficient (*r*, 95% CI). Effect sizes (ES) were calculated to interpret the magnitude of the mean difference between conditions with *d* < 0.2, *d* = 0.2–0.5, *d* = 0.5–0.8 and *d* > 0.8 considered as trivial, small, moderate and large, respectively (Cohen, 1988). Confidence intervals (95% CI) were used to specify estimation of changes in sleep and training load variables. The level of significance was set at *p* < 0.05.

## Results

The descriptive statistics for the athletes’ characteristics and sleep questionnaires are presented in Table 1. Seven of the 15 athletes reported a PSQI score =5, indicating poor overall sleep quality. Table 2 provides an overview of the daily monitored sleep variables and training load.

**TABLE 1:** Participants’ characteristics and general sleep measures.

**Athlete characteristic**	
Age (years)	21.7 (2.2)
Sex (n,% female)	8 (53%)
**Sleep questionnaires**	
PSQI (A.U)	5.7 (2.4)
**Chronotype**	
Morning types (%)	10 (67)
Intermediate types (%)	4 (27)
Evening types (%)	1 (6)

*Data are reported as mean (SD). PSQI, The Pittsburgh Sleep Quality Index.*

**TABLE 2:** Overview of the daily sleep variables and training load.

Bed time (hh:mm)	23:57 (23:12–00:30)
Get-up time (hh:mm)	07:52 (07:24–08:24)
Time in bed (min)	475 (450–489)
Total sleep time (min)	418 (380–445)
Wake after sleep onset (%)	9.5 (8.5–10.4)
Sleep efficiency (%)	88.3 (84.6–90.2)
Sleep onset latency (min)	10.0 (5.1–15.5)
Fragmentation index (A.U.)	28.5 (24.1–32.8)
SSI (A.U.)	21.2 (19.6–22.9)
Sleepiness (A.U.)	3.8 (3.4–4.2)
Daily s-RPE (A.U.)	692 (597–787)

*Data are reported as mean (95% CI). A.U., arbitrary unit; SSI, Spiegel Sleep Inventory; s-RPE, Rating of Perceived Exertion of the Session.*

**FIGURE 2:**
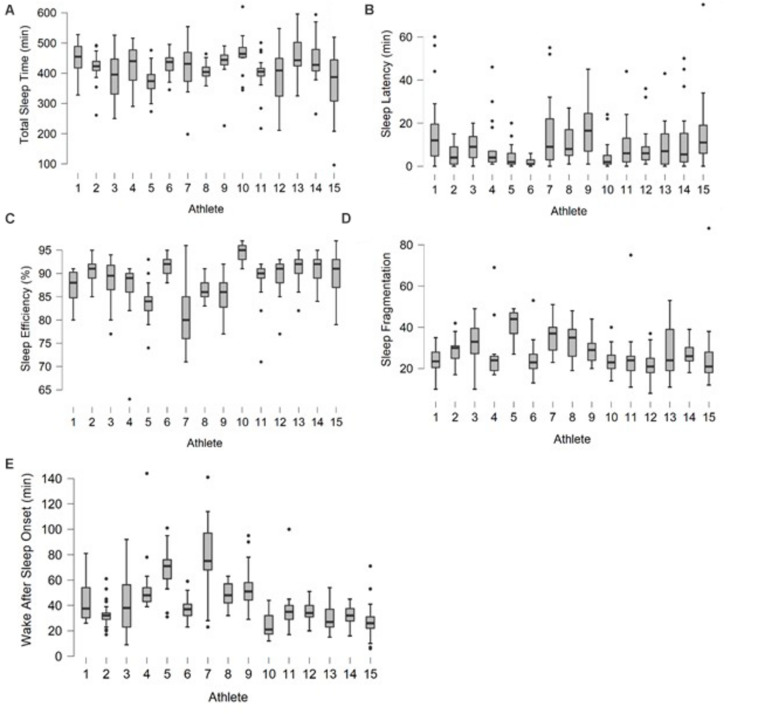
Individual athlete total sleep time **(A)**, sleep onset latency **(B)**, sleep efficiency **(C)**, sleep fragmentation **(D)** and wake after sleep onset **(E)** during the study period.

Figure 2 provides an overview of individual sleep variables during the study.

As a group, total sleep time ranged between 367 min [319; 416] and 457 min [425; 490] whereas total sleep time CV ranged between 7 and 34%. Sleep efficiency ranged between 80.7% [78.0; 83.5] and 91.5% [90.6; 92.4] whereas sleep efficiency CV ranged between 2 and 33%.

Three socio-physiological factors influencing sleep emerged from the qualitative interview analyses, i.e., sleep environment, training and study requirements (Figure 1).

### Sleep Environment

Based on the qualitative interviews, we were able to split the population into “complainers” (*N* = 8) and “non-complainers” (*N* = 7) of their sleeping environment. “Complainers” exhibited impaired sleep efficiency (87.6% ± 5.6 vs. 89.2% ± 4.3; *p* < 0.05; *d* = 0.31), WASO (47.4 min ± 23.9 vs. 37.5 min ± 17.1; *p* < 0.001; *d* = 0.45), SSI score (20.2 ± 4.1 vs. 22.5 ± 3.2; *p* < 0.001; *d* = 0.61) and level of sleepiness upon awakening (5.4 ± 2.0 vs. 4.4 ± 1.7; *p* < 0.01; *d* = 0.34) compared with the “non-complainers. “Complainers” reported later bedtime (00:03 ± 1:00 vs. 23:49 ± 1:12; *p* < 0.05; *d* = 0.21) and wake-up time (8:10 ± 1:06 vs. 7:30 ± 0:59; *p* < 0.001; *d* = 0.61) compared with the “non-complainers.” Noise and/or bedroom temperature and/or mattress quality and/or light exposure were presented as sleep environment issues by the “complainers”:

*”Noise is present, because I live on a busy part of campus. So I can even be disturbed at noon when I’m having a nap. It wakes me up, and I can also hear the neighbors.” Kate*

*”In summer, we all agree that it’s unbearably hot, and it makes it very difficult to sleep.” Emily*

*”The mattress is cheap, and so are the pillows. I have to bring my own, and that’s really not ideal.” Victoria*

*”The curtains cover half of the window. We are consistently exposed to outdoor lighting.” Neil*

The qualitative interviews allowed to split the population into athletes sleeping in a “shared bedroom” (*N* = 7) or a “single bedroom” (*N* = 8). The “shared bedroom” sub-group demonstrated impaired SSI score (20.9 ± 3.8 vs. 21.7 ± 3.8; *p* < 0.05; *d* = 0.31) and level of sleepiness upon awakening (5.6 ± 2.1 vs. 4.4 ± 1.5; *p* < 0.001; *d* = 0.32) compared with the “single bedroom” sub-group. One of the “shared bedroom” athletes postulated that a discrepancy in sleep-wake patterns may be the main reason of impaired sleep:

*”Sharing a room with someone is always more complicated than living alone, because we don’t have the same lifestyle patterns” Peter.*

Most of the athletes pertaining to the “shared bedroom” sub-group expressed a better sleep when occasionally sleeping alone:

*”I sleep better alone, I feel more free. I might go to bed later but I will be less tired” Charlotte*

Screen use within the hour preceding bedtime was reported in 63% of the nights studied, mainly for movies/series and/or social networking:

*”Unfortunately it’s often Netflix, even though I know it’s not ideal, but after a day of training it’s harder to open a book”. Peter*

For some athletes, their use lasted several hours:

*”It can easily be three hours of screen time before bedtime.” Mick.*

### Training

As a group, daily training load ranged between 352 A.U. [273; 430] and 1016 A.U. [901;1132] with a mean of 692 A.U. [597; 787]. Mean daily training volume ranged between 180 min [163; 197] and 420 min [397; 445] with a mean of 262 min [232; 396]. Daily training load showed correlations with WASO and sleep onset latency (*r* = 0.30 [0.25; 0.37] and *r* = 0.20 [0.17;0.26], *p* < 0.05, respectively). The results of the qualitative interviews aimed to go beyond these figures and explain the reasons for players’ impaired sleep in relation to training. Some athletes were required to attend an early morning training session (7:00 a.m.) 1 day per week which represents some challenges:

*”It’s hard to get out of bed” Mick*

*”Yeah, it’s clear that I’m not in the same state” Tom*

The interview findings demonstrated that seven of the athletes (i.e., 47%) reported sleeping alterations after training sessions perceived as “hard”:

*”In fact, the more intense the training, the heavier my legs are going to feel, I’ve already woken up at night because of that, muscular soreness, hot and heavy legs.” Victoria.*

### Study Requirements

The qualitative interviews allowed to split the population into “student athletes” (*N* = 10) and “non-student athletes” (*N* = 5). “Student athletes” exhibited later bedtime (23:58 ± 1:06 vs. 23:45 ± 0:52; *p* < 0.05; *d* = 0.33) and earlier wake-up time (07:44 ± 0:55 vs. 07:55 ± 0:35; *p* < 0.05; *d* = 0.32) compared with “non-student athletes.” “Student athletes” demonstrated impaired total sleep time (6:50 ± 1:13 vs. 7:06 ± 0:53; *p* < 0.05; *d* = 0.20), WASO (47 min ± 21 vs. 39 min ± 21; *p* < 0.001; *d* = 0.49), SSI score (20.6 ± 4.0 vs. 22.1 ± 2.7; *p* < 0.01; *d* = 0.30) and level of sleepiness upon awakening (5.3 ± 2.0 vs. 4.6 ± 1.0; *p* < 0.05; *d* = 0.31) compared with “non-student athletes.” “Student athletes” attended 13.2 ± 6.6 classroom hours per week. Interviews with “student athletes” revealed some of the difficulties athletes had in conciliating training and studying:

*”Yeah, but that’s the way it is, when you take a look at my schedule, there’s not a moment in the day when I can rest, I start at 8 a.m. and finish at 7 p.m., I love what I do, there’s no discussion about that, ideally, I should study in the evening but I don’t because frankly it’s not possible, I’m too tired*…*I sometimes even fall asleep in class, for example last Monday.” Peter*

*”It’s really hard for me to focus on the lessons, not because I’m tired, but because I don’t like being imposed a certain schedule, and having to learn at specific times. So it’s true that when I feel tired, I don’t listen to anything and I go back to it later when I really feel able to learn, otherwise it makes me even more tired.” Victoria*

## Discussion

The present study allowed for an examination of sleep-wake behavior among elite athletes. In addition to objective sleep assessment, qualitative interviews were carried out to investigate athletes’ sleeping patterns as well as the socio-physiological acute and chronic stressors affecting their sleep. Leeder et al. (2012) suggested that as good practice, reference data for “normal” athlete sleep behaviors measured in their own homes should be obtained, to allow for comparisons with heavy training load and competing schedules. The elite (Olympic) athletes they studied spent on average 516 min in bed and slept 415 min with a high inter-individual variability in sleep measures. In this respect, it may be important to include the presentation of sleep data by encompassing individual responses, in addition to general group means. In the present study, elite athletes spent approximately 40 min less in bed, but achieved on average a similar total sleep time, i.e., ~7 h of sleep per night. All athletes presented good sleep efficiency (88.3%; individual range between 80.7 and 91.5%). Total sleep time CV ranged between 7 and 34% whereas sleep efficiency CV ranged between 2 and 33%, which confirms both high intra- and inter-individual variability of sleep among elite athletes (Costa et al., 2019). Even if 7 of the 15 athletes reported poor overall sleep quality (i.e., PSQI score ≥ 5), daily monitored actigraphic measures revealed that sleep efficiency—a sensitive metric for estimating sleep quality—was 88 ± 5%. However, this data masks high variability in individual sleep. To the best of our knowledge, the present study is the first to use a mixed-method approach combining objective assessment with qualitative interviews, in order to encompass specific socio-physiological factors influencing the sleep of athletes. Three main factors emerged from the qualitative interview analyses, i.e., sleep environment, training and study requirements.

As previously reported, it is virtually impossible to take all of the influencing factors into consideration when studying the sleep of a homogeneous group of elite athletes (Sperlich and Holmberg, 2017). In terms of implementation, there is a need to take into account the broad ecological context of the sporting environment, including identifying intrapersonal factors, sociocultural factors, policies, and the physical environment (Joncheray et al., 2019). In this respect, the methodology of qualitative interview may be suitable, especially to address factors which are currently underreported among athletes. Here, half of the population (8/15) complained of their sleeping environment. Factors such as noises inside/outside the room, bedroom temperature, mattress quality and/or light exposure were identified as potential reasons for poor sleep in “complainers” which have been confirmed elsewhere (Cajochen, 2007; Kräuchi, 2007; Griefahn et al., 2008; Aloulou et al., 2020). “Complainers” notably exhibited impaired sleep efficiency and WASO compared with “non-complainers.” A lower sleep efficiency index is one of the features of the first-night effect, thought to result from an individual’s lack of adaptation to the sleeping environment (Suetsugi et al., 2017). As the athletes involved in the study often change their sleeping environment throughout the season, requiring a regular adaptation to the “normal” one, future studies may assess the effectiveness of environmental sleep hygiene strategies (e.g., mattress; Aloulou et al., 2020) to chronically improve athletes’ sleeping measures.

Several studies have assessed the influence of training load on sleep. In the present study, daily training load showed small correlations (*p* < 0.05) with WASO and sleep onset latency but not with total sleep time and sleep efficiency. In different settings, other authors have revealed no evidence for adaptations in sleep quantity/quality following day-to-day variation in training load (Knufinke et al., 2018; Costa et al., 2019). The results of the present qualitative interviews aimed to explain the reasons for athletes’ impaired sleep in relation to training. The common practice of early morning training sessions (7:00 a.m.) posed some challenges to the athletes in the present study. Accordingly, it may be associated with a reduction in total sleep, higher daytime sleepiness, and poorer sleep quality compared to training during the day (Swinbourne et al., 2016). Athletes also reported sleep alterations after training sessions perceived as “hard,” possibly because of pain and movements during sleep. However, the influence of exercise-induced delayed onset muscle soreness on sleep quantity/quality is still debated (Hausswirth et al., 2014). Future studies are also required to explore the reciprocal influence between day-to-day variations in perceived training load and adaptations in sleep quantity/quality among elite athletes (Knufinke et al., 2018).

Practicing a sport at the highest level involves high individual pressure with plenty of stressors, and constraints on one’s personal life (Schaal et al., 2011). One of the main challenges athletes face is combining elite sport with other pursuits such as education and/or work (Burlot et al., 2018). In the present study, “student athletes” exhibited later bedtime and earlier wake-up time compared with “non-student athletes,” leading to a reduced total sleep time (6:50 ± 1:13 vs. 7:06 ± 0:53; *p* < 0.05). It was reported that late bedtime, caused by lifestyle, social activity, and phase delays of the circadian pacemaker, combined with early study start times, can make achieving the recommended sleep duration challenging (Crowley et al., 2018), especially for student athletes. Even if not tested in the present study, training and school schedules which reduce total sleep time can also have a significant impact on sleep structure (Miller et al., 2014). Future studies may assess the effectiveness of altering the athletes’ academic and training schedules around an extended sleeping period to increase sleep time, especially for student athletes.

Some methodological aspects of the present study must be considered. All athletes in the present study were involved in individual sports. Team sports may offer specific factors in addition to those investigated in the present study, e.g., late night matches. Other socio-physiological factors influencing the sleep of athletes anecdotally emerged from the qualitative interview analyses and might be worth expanding on, at least from an individual perspective. There is currently not available data on the validity of the Spiegel Sleep Inventory whereas baseline mean values (e.g. during pre-season) were not established in the present study.

## Practical Applications

The present study highlights the substantial individual variability in sleep among elite athletes suggesting the adoption of an individual approach to monitor sleep. The addition of a qualitative interview to the common sleep analysis tools may provide a better understanding of individual sleep-wake behavior. It may be viewed as a promising strategy not only to help identify individuals at risk but also to improve sleep, recovery and performance during highly demanding training periods.

## Conclusion

The present investigation was conducted as a real-world applied example for athletes and practitioners seeking to deploy sleep hygiene strategies to maximize performance. A better understanding of the socio-physiological stressors affecting the athletes’ sleeping patterns—sleep environment, training and study requirements—may be of direct benefit to enhance recovery, and preparations for subsequent training and competition. Therefore, sport scientists may individually assess sleep and sleep hygiene using actigraphy and interviews. Based on this approach, individualized sleep interventions with consideration to physiological (e.g., training load), behavioral (e.g., screen use) and environmental factors (e.g., room temperature, noise, mattress), is then recommended.

## Data Availability Statement

The raw data supporting the conclusions of this article will be made available by the authors, without undue reservation.

## Ethics Statement

The studies involving human participants were reviewed and approved by East III, France. Ref. 170605. The patients/participants provided their written informed consent to participate in this study.

## Author Contributions

KB, HJ, and MN designed the research, conducted the experiments, and wrote the manuscript. KB, JE, CL, HJ, and MN analyzed and interpreted data and approved the final version of the manuscript. All authors contributed to the article and approved the submitted version.

## Conflict of Interest

The authors declare that the research was conducted in the absence of any commercial or financial relationships that could be construed as a potential conflict of interest. The handling editor is currently editing co-organizing a Research Topic with one of the authors.

## Publisher’s Note

All claims expressed in this article are solely those of the authors and do not necessarily represent those of their affiliated organizations, or those of the publisher, the editors and the reviewers. Any product that may be evaluated in this article, or claim that may be made by its manufacturer, is not guaranteed or endorsed by the publisher.
